# UNDERSTANDING THE ROLE OF SOCIAL CAPITAL IN FORMAL SERVICE USE AMONG OLDER ADULTS: A SCOPING REVIEW

**DOI:** 10.1093/geroni/igae098.1829

**Published:** 2024-12-31

**Authors:** Carson De Fries, Kaipeng Wang, Leslie Hasche

**Affiliations:** University of Denver, Denver, Colorado, United States; University of Denver, Denver, Colorado, United States; University of Denver, Denver, Colorado, United States

## Abstract

As individuals age, they may encounter new or persistent challenges that affect their need for and access to formal services. Social capital theory, which views relationships as a source of support, a bridge to needed resources and information, and a buffer against challenges that individuals may face, serves as a useful framework in addressing barriers to service utilization among this population. This scoping review aimed to identify how an older adult’s social capital influences their use of formal services, with a focus on the differences between intergenerational and peer (i.e., non-intergenerational) relationships. The search, conducted in January of 2023, originally yielded 2,645 articles. Following duplication removal and eligibility screening, 57 articles were included for analysis. In reviewing the methodologies of these articles, 75.4% (n=43) used quantitative methods, 9.5% (n=5) utilized qualitative methods, 3.8% (n=2) conducted a systematic or scoping review, one used mixed methods, and one was a conceptual paper on the topic. Results demonstrate nuanced differences in how certain types of relationships may support older adults’ access to formal services. Bonding social capital, operationalized as peer relationships, often provided more therapeutic support, which may have reduced the need for formal services. Bridging social capital, operationalized as intergenerational relationships, often resulted in more instrumental support, providing older adults with information about diverse resources and services. Findings also highlighted methodological gaps in assessing older adults’ social relationships and underscored the need to further explore the mechanisms through which different relationship types influence older adults’ access to needed care.

